# Simplifying the Diagnosis of Vertical Diplopia: Is It Skew or Not?

**DOI:** 10.3390/jemr19020037

**Published:** 2026-04-08

**Authors:** Anas Igbariye, Noa Hadar, Basel Obied, Adi Berco, Alon Zahavi, Inbal Man Peles, Nitza Goldenberg-Cohen

**Affiliations:** 1 Department of Ophthalmology, Bnai Zion Medical Center, Haifa 3339419, Israel; anasigba@gmail.com (A.I.); noahadar78@gmail.com (N.H.); adiberco@gmail.com (A.B.);; 2 The Krieger Eye Research Laboratory, Faculty of Medicine, Technion–Israel Institute of Technology, Haifa 3339419, Israel; basel.obied01@gmail.com; 3 Department of Ophthalmology and Laboratory of Eye Research, Felsenstein Medical Research Center, Rabin Medical Center, Petach Tikva 4941492, Israel; alonzahavi@gmail.com; 4 Gray Faculty of Medical & Health Sciences, Tel Aviv University, Tel Aviv 6997801, Israel

**Keywords:** trochlear nerve palsy, ocular tilt syndrome, skew, cyclotorsion, fundus camera

## Abstract

Ocular tilt reaction (OTR) and trochlear nerve palsy (TNP) can induce cyclotorsion. We aimed to assess the utility of fundus photography in distinguishing between these disorders. The database of a neuro-ophthalmology hospital-based clinic was retrospectively searched for patients referred for new-onset vertical diplopia between 2020 and 2023. Medical data were collected, and the angle between the optic disc and fovea was measured using ImageJ software to quantify torsion. Distinct torsional patterns were identified between the groups. OTR was characterized by variable, often conjugate torsion, whereas TNP demonstrated consistent disconjugate extorsion. Analysis of interocular torsional relationships, rather than magnitude alone, provided useful diagnostic discrimination. Fundus photography may be useful for differentiating OTR from TNP in complicated neurological cases, particularly in patients who are difficult to examine. This study emphasizes the practical clinical value of fundus photography as a simple, accessible, and objective tool for differentiating OTR from TNP, by contributing the torsional component of OTR triad, particularly in emergency or diagnostically challenging settings where standard examination may be limited.

## 1. Introduction

Ocular tilt reaction (OTR) is a vertical misalignment of the eyes caused by damage to prenuclear vestibular input to the ocular motor nuclei. OTR is primarily a brainstem sign, but can be seen with acute unilateral cerebellar lesions as well [1,2]. OTR is usually accompanied by conjugate ocular torsion toward the same side, head tilt, and subjective vertical deviation of the visual scene [1]. The typical cyclotorsional pattern in OTR is intorsion of the hypertropic eye, whereas in trochlear nerve palsy (TNP), it is extorsion of the hypertropic eye; exceptions may occur.

Ocular torsion reflects the static component of the three-dimensional ocular motor system and is closely linked to vestibular and otolithic input [3,4]. From an eye movement research perspective, torsion represents the torsional component of Listing’s law—constrained eye position—and is modulated by central vestibular pathways [5]. Thus, OTR and TNP reflect distinct ocular motor control mechanisms: OTR produces a conjugate torsional bias driven by vestibular imbalance, whereas TNP produces a disconjugate torsional deviation resulting from selective extraocular muscle dysfunction. Quantitative characterization of torsional eye position has been investigated using scleral search coils, video-oculography, and fundus-based anatomical landmarks [6,7,8]. However, these techniques are not routinely available in acute clinical settings.

It is difficult to differentiate OTR from TNP, also referred to as superior oblique palsy or fourth nerve palsy. The hint to the correct diagnosis is related to bilateral or monocular cyclotorsion characteristic of each disorder [9]. Fundus photography can assist in the evaluation of ocular torsion and support the diagnosis [9]. The disc–fovea angle can be used as an indicator in cyclovertical torsion [10]. Minimal disc–fovea angle variation on triplicate repeated photos can be considered an accurate objective measurement of ocular torsion [10]. The disc–fovea angle provides an anatomical surrogate for static torsional eye position relative to retinal landmarks and serves as a reproducible structural correlate of ocular alignment.

Identifying the underlying cause of visual disturbances is crucial, particularly in severely ill and difficult-to-examine patients. A straightforward, rapid, and precise method is warranted. Fundus photos offer a simple means to document and analyze ocular torsion, using either a non-mydriatic fundus camera or optical coherence tomography (OCT) examination with the glaucoma software analysis module [11]. Unlike dynamic eye movement recording systems, fundus photography captures the static torsional state, which may nonetheless reflects the underlying vestibular or muscle-specific imbalance [12].

The aim of this study was to simplify differentiation between OTR and TNP by quantitatively assessing static torsional eye position using fundus-derived disc–fovea angles. Non-mydriatic fundus photography is readily accessible and does not require a specialized technician. The photos can be analyzed immediately, even in the emergency room. By translating torsional eye-position quantification into routine clinical practice, this approach may bridge laboratory-based eye-movement research and bedside neuro-ophthalmologic assessment. Additionally, in the future, the images might be analyzed by artificial intelligence and deep learning systems.

## 2. Materials and Methods

### 2.1. Study Cohort

A retrospective study design was used. Medical records and fundus photos of patients referred to a neuro-ophthalmology hospital-based clinic with new-onset vertical diplopia from January 2020 to September 2023 were reviewed. Data were collected on patient demographics, medical history, clinical symptoms and signs, findings on neurological and ophthalmological examinations, and final diagnosis. Both neuroimaging data and fundus photographs were analyzed. Patients with alternative causes of vertical diplopia, including third cranial nerve palsy, myasthenia gravis, thyroid eye disease, and restrictive or mechanical etiologies, were excluded based on clinical evaluation, ancillary testing, and neuroimaging when indicated. Only patients with a final diagnosis of ocular tilt reaction (OTR) or trochlear nerve palsy (TNP) were included in the analysis.

In addition to standard neuro-ophthalmologic evaluation, the analysis quantified static torsional eye position using retinal anatomical landmarks, thereby enabling objective characterization of ocular motor alignment patterns.

This study was conducted in accordance with the Declaration of Helsinki, and the protocol was approved by the Ethics Committee of Bnai Zion Medical Center (BNZ-0012-23 on 23 February 2023).

### 2.2. Torsion Measurements

Fundus photographs were captured using the CenterVue non-mydriatic fundus camera DSplus (CenterVeu S.p.A, Padova, Italy). Each eye was investigated separately in the primary position. Primary position was defined as fixation on the camera’s internal target, minimizing voluntary gaze deviation and approximating habitual eye position used in static ocular motor research paradigms.

**Figure 1:**
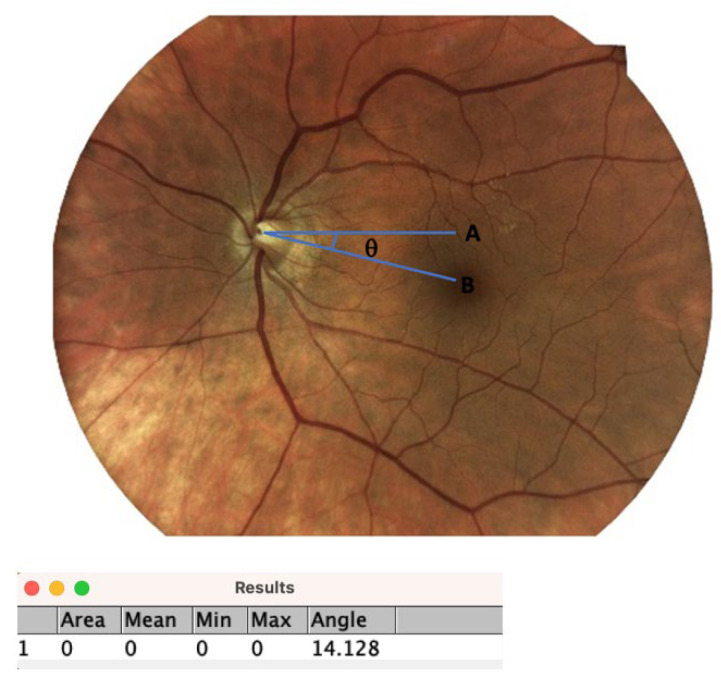
Disc-center–fovea angle measurement. Measurement of the disc–fovea angle using ImageJ is illustrated. A = horizontal line drawn from the optic disc center; B = line drawn from the optic disc center to the fovea. θ = the angle between lines A and B.

Photos were analyzed for torsion with ImageJ software (ImageJ 1.54f, National Institute of Health, Bethesda, MD, USA) [7,13]. The angle formed between a line passing through the center of the optic disc to the fovea and a horizontal line passing through the center of the disc (disc–fovea angle) was calculated using fundus photographs (Figure 1) [13].

The disc–fovea angle served as an anatomical surrogate for static torsional eye position relative to retinal landmarks, analogous to torsional position metrics obtained in laboratory-based eye movement studies using scleral search coils or video-oculography systems [6]. Unlike dynamic eye tracking methods, this approach quantifies the structural correlate of ocular alignment at a fixed time point.

The examiner manually identified the midpoint of the optic disc and used it as a reference point. The foveal location was determined based on the fixation target provided by the fundus camera system (visible as a green spot), corresponding to the center of fixation. All measurements were performed independently by two trained observers to enhance consistency and reduce subjective bias.

Interobserver agreement was assessed to ensure reproducibility of torsional eye position measurements, consistent with methodological standards in quantitative eye movement research.

Cyclotorsional movements were quantified, with extorsion as a positive value and intorsion as a negative value. The absolute total extorsion value and the sum of deviations of both eyes were calculated. Overall ocular torsion was defined as the sum of the disc–fovea angles in the two eyes in cases of bilateral extorsion.

Torsional patterns were analyzed in terms of directionality and conjugacy to distinguish vestibular-driven conjugate torsion from disconjugate torsion associated with extraocular muscle paresis.

### 2.3. Statistical Analysis

Statistical analyses were performed using GraphPad Prism software (version 10.2). Due to the small sample size and non-normal distribution of the data, differences between groups were evaluated using the nonparametric Mann–Whitney U test. Categorical variables were analyzed using the Chi-squared test. Effect sizes were calculated using rank-biserial for continuous variables. A two-sided *p*-value ≤ 0.05 was considered statistically significant.

Statistical comparisons focused on differences in torsional magnitude and conjugacy patterns between diagnostic groups, reflecting distinct ocular motor control mechanisms.

## 3. Results

A total of 32 patients were included in the study, 8 with OTR and 24 with TNP (20 non-traumatic and 4 traumatic). All patients presented with vertical diplopia, except for one congenital case with suppression, in whom diplopia was not reported. In this patient, the vertical deviation was subtle and not clearly detected on cover testing, but fundus photography demonstrated intorsion and suggested relative vertical misalignment between the eyes, supporting inclusion in the OTR group. The demographic characteristics, vascular risk factors, and underlying etiologies of the cohort are shown in Table 1.

A summary of statistical comparisons between OTR and TNP groups is presented in Table 2. No significant differences in demographic data were detected between the OTR and the TNP groups. The male-to-female ratio was 3:1 in the OTR group and 5:3 in the TNP group. Vascular risk factors in both groups included hypertension, diabetes mellitus, ischemic heart disease, and hypercholesterolemia. Some patients had multiple risk factors. The OTR and TNP etiologies identified in the patients are shown in Table 1. Only one patient in the TNP group had a confirmed cerebrovascular event (parieto-occipital infarction) in the past, which was likely unrelated to the TNP. The remaining patients did not have documented stroke, but had microvascular risk factors consistent with presumed ischemic isolated cranial nerve palsy. Demographic variables did not differ significantly between groups, suggesting that differences in torsional measurements were unlikely to be attributable to baseline systemic factors.

Among the eight patients diagnosed with OTR, five demonstrated intorsion of the hypertropic eye, while one patient showed isolated intorsion without associated vertical deviation (Table 3). The remaining two patients exhibited extorsion in the hypertropic eye while the other eye was intorted. Overall, torsional patterns in the OTR group were predominantly conjugate in direction, consistent with vestibular-driven ocular motor imbalance. Notably, these two had complex neurological histories: one underwent neurosurgery for pilocytic astrocytoma resection and presented with left hypertropia and extorsion (with bilateral leftward torsional eye movements), while the other sustained severe head trauma with multiple orbital fractures and exhibited right hypertropia and extorsion. These two cases were initially misdiagnosed as TNP; however, fundus photography revealed intorsion in the fellow eye, supporting a final diagnosis of OTR. In these cases, analysis of torsional conjugacy, rather than torsional magnitude alone, enabled differentiation between central vestibular imbalance and isolated extraocular muscle paresis.

In the OTR group, the 3-step test was completed in 7/8 patients and yielded positive results in all 5. One of these patients also underwent the 4th step (upright-supine test), which was likewise positive. In one child with congenital OTR, the test was negative. Limited cooperation may have reduced the reliability of subjective ocular alignment testing in this case. In the two remaining cases, the test was not performed—one due to the absence of vertical deviation and the other due to the patient’s poor general medical condition.

In the TNP group, the 3-step test was performed in all 24 patients. The results were positive in 22, inconclusive in 1, and negative in 1. The latter had a complex strabismus history, including prior right inferior oblique myectomy and right superior rectus recession, which may have affected the reliability of the test outcomes. Torsional measurements in the TNP group demonstrated predominantly disconjugate extorsion patterns, consistent with selective superior oblique muscle dysfunction rather than vestibular imbalance.

**Figure 2:**
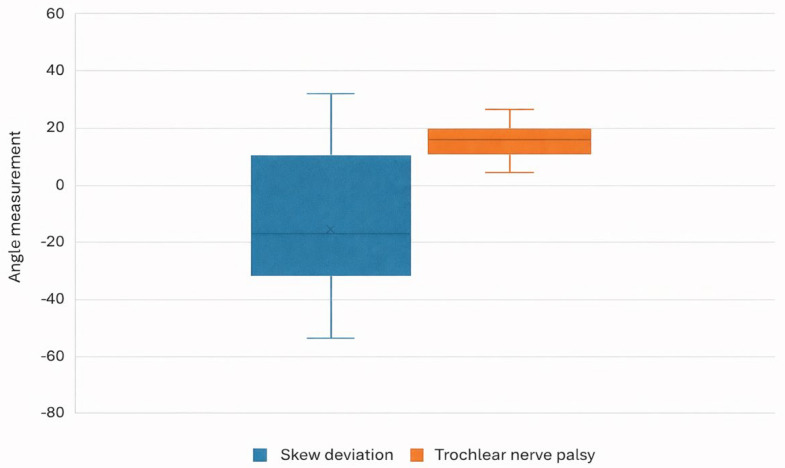
Variability of disc–fovea angle measurements. Boxplot showing disc-angle measurements in degrees in patients with OTR or TNP. The boxplot demonstrates wider dispersion of torsional directionality in OTR compared to the relatively uniform positive torsional distribution observed in TNP.

**Figure 3:**
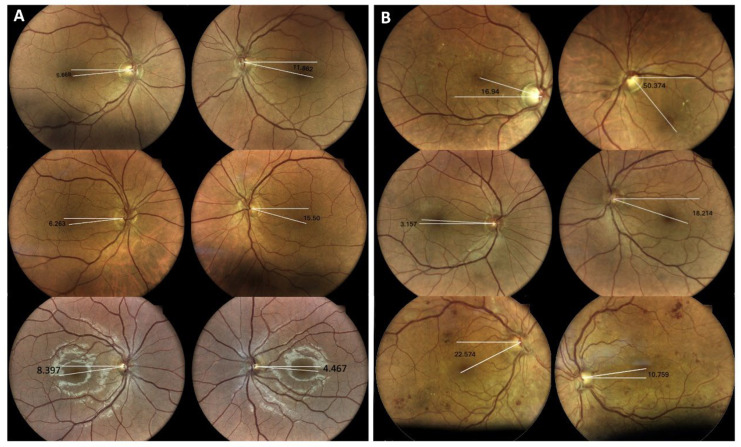
Representative disc–fovea angle measurements in OTR and TNP. (**A**) Three patients with TNP; (**B**) three patients with OTR. Representative cases illustrate the contrast between disconjugate extorsion in TNP and conjugate torsional deviation in OTR. Vertical deviation is also evident. In OTR, the hypertropic eye demonstrates intorsion; however, due to the mirrored presentation of the fundus images, this may appear as if the hypotropic eye is intorted.

The mean disc–fovea angle was calculated for the OTR and TNP groups. The sum of intorsion + extorsion in the OTR group (which reflects net directionality) was −11.25 ± 10.56°, and the absolute total value (which reflects overall torsional magnitude regardless of direction) was 25.48 ± 19.47°. The sum of extorsion and total absolute in the TNP group were identical and calculated as 23.23 ± 9.79° (*p* < 0.0001) (Table 3 and Table 4 and Figure 2 and Figure 3). While the overall torsional magnitude overlapped between groups, the directionality and interocular relationship differed substantially. The OTR group demonstrated greater variability in net torsional direction, reflecting conjugate vestibular bias, whereas the TNP group exhibited consistently positive torsional values corresponding to disconjugate extorsion. These findings indicate that torsional pattern analysis, rather than magnitude alone, provides discriminative value in distinguishing central from peripheral ocular motor mechanisms.

## 4. Discussion

This retrospective study highlights the clinical utility of non-mydriatic fundus photography as a precise, objective, and noninvasive tool for differentiating OTR from TNP, particularly in diagnostically complex cases involving cerebrovascular pathology. While fundus photography has previously been described for assessment of ocular torsion, the novelty of this study lies in its emphasis on real-world clinical application as an accessible and objective diagnostic adjunct. In particular, fundus imaging enables identification of torsional patterns, including intorsion of the hypertropic eye, and may support the diagnosis of OTR and help in completing the diagnostic triad, as commonly torsion is the missing component in the diagnosis, especially in acute or neurologically complex presentations. From an eye movement research perspective, fundus-based analysis enables quantification of static torsional eye position and permits characterization of interocular torsional relationships. While fundus photography reliably quantifies ocular torsion, its diagnostic value may be limited in isolation and should ideally be interpreted alongside other clinical signs, such as nystagmus or vertigo.

Although the etiologies in our cohort were diverse, the small number of OTR cases and retrospective study design preclude firm conclusions regarding etiological distinctions between groups. Nonetheless, the findings emphasize the role of fundus photography in recognizing torsional patterns that can support diagnostic decision-making in challenging cases. Importantly, the distinction between conjugate vestibular-driven torsion and disconjugate muscle-palsy torsion reflects fundamentally different ocular motor control mechanisms [14,15]. In OTR, torsion represents a supranuclear phenomenon arising from imbalance of the utriculo-ocular and graviceptive vestibulo-ocular reflex pathways in the roll plane. Lesions involving the vestibular nuclei, medial longitudinal fasciculus, or interstitial nucleus of Cajal produce a tonic roll-plane bias that affects both eyes in a coordinated manner, generating conjugate torsion toward the hypotropic eye and often accompanying head tilt and deviation of subjective visual vertical [14]. In contrast, trochlear nerve palsy reflects selective dysfunction of a single extraocular muscle, resulting in monocular or disconjugate extorsion of the paretic hypertropic eye without a global roll-plane vestibular imbalance [15]. Thus, OTR reflects disruption of central vestibular integration within brainstem pathways, whereas trochlear palsy reflects peripheral muscle-specific weakness, leading to fundamentally different patterns of interocular torsional coupling. While TNP is often associated with trauma and congenital anomalies, it can also arise from stroke, etiologies that similarly contribute to OTR [16,17].

Differentiating TNP from OTR remains clinically challenging, as both may present with cyclotorsion and acute vertical diplopia [16]. The three-step (Parks–Bielschowsky) test is a clinical method used to localize vertical strabismus by assessing changes in hypertropia with gaze direction and head tilt, thereby helping to identify involvement of specific extraocular muscles, particularly in suspected trochlear nerve palsy. The three-step test is conventionally used to distinguish these conditions; however, it was positive in five out of eight OTR patients in our cohort, and the fourth “upright-supine” step, as described by Wong et al. [18], was performed in only one case. The three-step test indirectly infers an extraocular muscle imbalance, whereas fundus-based torsional analysis directly quantifies ocular alignment relative to retinal landmarks. In TNP, excyclotorsion typically occurs in the affected eye, whereas in OTR, intorsion is more often seen in the hypertropic eye. This finding, while not absolute, offers higher specificity. In our study, two patients initially presumed to have TNP were ultimately diagnosed with OTR after fundus imaging revealed extorsion in the hypertropic eye and compensatory intorsion in the contralateral hypotropic eye. Such patterns, particularly when occurring in conjugate torsion to the same side, may suggest OTR in cases with overlapping features. These cases underscore that torsional conjugacy, rather than torsional magnitude alone, provides key insight into the underlying ocular motor mechanism. Notably, patients with a history of craniotomy present an additional diagnostic challenge, as the surgical intervention may induce TNP; however, the underlying neurological insult, such as tumor resection or trauma, can itself result in OTR [18].

Given these diagnostic complexities, fundus photography serves as a valuable adjunct to the three- or four-step test. In our experience, it proved helpful in differentiating skew deviation from superior oblique palsy, particularly in acutely ill patients who could not cooperate with standard motility examinations. Our findings align with prior reports demonstrating that fundus photography enables quantification of the disc–fovea angle and facilitates objective assessment of cyclotorsion [7,19]. In typical OTR, the hypertropic eye demonstrates intorsion, while the hypotropic eye may show extorsion. Not all patients in our cohort demonstrated the complete OTR triad of vertical deviation, ocular torsion, and head tilt. This likely reflects both the known variability in clinical presentation and the retrospective nature of the study, which may limit documentation of all components. Vertical diplopia was present in all but one congenital case with suppression, in whom only a subtle vertical deviation was suspected clinically, but was supported by fundus findings. Ocular torsion, identified by fundus photography, was the most consistently documented feature and served as a key diagnostic element in all cases, whereas head tilt was recorded in only a minority of patients. These findings underscore that OTR may present with incomplete clinical features, and highlight the value of fundus-based torsion assessment in supporting the diagnosis, particularly in ambiguous or diagnostically challenging cases. However, complex neuroanatomical disruption may yield atypical patterns [20].

Three notable cases in our cohort required diagnostic reconsideration after fundus photography: two adults with a history of brain injury or tumor surgery and one child with isolated torsion and no vertical deviation. In all three cases, fundus imaging demonstrated intorsion of the hypotropic eye, with conjugate torsion towards the same side, supporting a final diagnosis of OTR. The child actually had vertical deviation but not diplopia due to suppression. Although this reevaluation refined the diagnostic understanding, it did not alter immediate clinical management. In congenital cases, imaging of an intact superior oblique muscle further supported a non-TNP etiology.

Various methods have been proposed to assess cyclotorsion. The double Maddox rod test allows subjective assessment of torsional misalignment [21], while newer technologies, such as eye-tracking systems [22] and smartphone-based applications [21], offer objective alternatives. Quantitative analysis of disc–fovea angle using calibrated fundus images has also been validated, with normal macular positioning approximately 0.20 ± 0.17 disc diameters above the horizontal disc line [21]. When this spatial relationship is disrupted, objective deviation, whether intorsion or extorsion, can be quantified, aiding diagnosis in both OTR and TNP [19].

In laboratory-based eye movement research, ocular torsion has traditionally been quantified using scleral search coil systems, which provide high-resolution recordings of three-dimensional eye position with high temporal and spatial resolution. More recently, validated three-dimensional video-oculography systems have enabled noninvasive measurement of dynamic torsional position and velocity by tracking stable iris or limbal features, allowing detailed analysis of vestibularly driven and head movement-related torsion under controlled experimental conditions [6]. In contrast, clinical assessment of torsion relies on objective anatomical landmarks, most commonly the disc center–fovea angle measured on fundus photography, which is considered the gold standard for static torsion quantification [7]. Optical coherence tomography with infrared imaging permits measurement under near-natural viewing conditions and demonstrates strong agreement with conventional fundus photography [7]. By comparison, wide-field fundus photography may systematically overestimate extorsion because of optical barrel distortion and alignment-related factors [7]. However, such systems are rarely available in routine clinical settings. In contrast, fundus photography captures the static torsional state at a defined fixation point. While this approach does not provide temporal dynamics, it offers a reproducible structural correlation of ocular alignment that can be readily implemented in clinical practice.

Although our study did not incorporate artificial intelligence (AI) or deep learning, the consistent measurement of disc–fovea angles provide a foundation for future automated diagnostic platforms. Manual disc–fovea angle (DFA) measurement has been shown to be both limited in reproducibility and time-consuming, with reported average errors of around 2.0° and variability between repeated measurements [23]. In contrast, fully automated deep learning-based segmentation approaches can localize the optic disc and macular centers and compute DFA directly from fundus images, achieving substantially lower average angular errors, as low as 0.76° using DeepLabv3+ [23]. More specifically, semantic segmentation networks such as DeepLabv3+ can accurately segment the optic disc and virtual macular regions, determine their centroids using geometric operations, and compute DFA using coordinate-based trigonometric computations [23]. In addition, dedicated deep learning frameworks have demonstrated precise automatic localization of the optic disc and macular centers with small centroid errors and high intersection-over-union and pixel accuracy metrics, enabling fully automated DFA calculation with sub-degree mean error [12,23]. Therefore, standardized retinal landmark detection and reproducible DFA quantification, as implemented in our study, are conceptually aligned with and readily adaptable to future AI-based automated torsion assessment systems.

OTR remains underreported in pediatric populations, potentially due to its rarity or the difficulty of examining neurologically impaired children. Most pediatric cases of OTR are described following posterior fossa tumor resection and tend to resolve spontaneously [24]. In our cohort, one child presented with OTR years after PA surgery, and another had idiopathic OTR without prior intervention. These findings underscore the need for improved recognition and documentation of torsion in children, as underestimation may lead to suboptimal surgical planning, especially in cases with poor response to standard strabismus surgery. Objective torsional quantification may be particularly valuable in pediatric patients with limited cooperation for dynamic eye movement testing.

Despite its clinical importance, ophthalmoscopy is underutilized in acute care settings due to the need for pharmacologic dilation and examiner expertise [25]. There is growing demand for accessible retinal imaging modalities, such as OCT and fundus cameras, in primary and emergency care [26]. Non-mydriatic fundus photography has proven effective in detecting urgent neurologic and ophthalmologic conditions, even in resource-limited environments [27]. It offers particular advantages in patients unable to cooperate with full neurological or ocular examination, such as children or those with impaired consciousness [28,29]. The FOTO-ED study demonstrated that non-mydriatic imaging can alter clinical management by revealing critical fundus findings [26].

While the general utility of non-mydriatic fundus photography is well-established, its specific application in diagnosing OTR has received limited attention. Our results support its role in differentiating OTR from TNP through objective measurement of disc–fovea angles. In typical OTR, the hypertropic eye exhibits intorsion. In certain cases, the hypotropic eye may show intorsion while the hypertropic eye demonstrates extorsion. These patterns contrast with the disconjugate excyclotorsion commonly seen in TNP. This distinction reflects the difference between central vestibular imbalance and isolated peripheral muscle dysfunction within the ocular motor system. This approach may expedite diagnosis even in critically ill patients [27,30].

This study was designed to evaluate the diagnostic contribution of fundus photography in cases of vertical diplopia from the ophthalmologist’s perspective. Systemic or neurologic correlates of vestibular dysfunction, such as vertigo or nystagmus, were not systematically analyzed and may provide complementary information in future studies. A further limitation was the incomplete three-step testing in one OTR patient.

Although individual torsional values may overlap between groups, the overall torsional pattern, including directionality, asymmetry, and its relationship to hypertropia, can offer diagnostic insight. Analysis of torsional conjugacy and directionality provides mechanistic information regarding the underlying ocular motor control system. When combined with clinical history, neuroimaging, and other ocular findings, fundus photography enhances diagnostic precision and may influence management in complex presentations. In several of our cases, torsional data obtained via fundus photography led to a reclassification of the underlying disorder, despite initial assumptions based solely on etiology or the clinical exam.

## 5. Conclusions

This retrospective study supports the clinical value of non-mydriatic fundus photography as a practical, noninvasive, and accessible tool for the objective assessment of cyclotorsion. From an ocular motor perspective, fundus-based disc–fovea angle analysis enables quantification of static torsional eye position and characterization of interocular torsional relationships. By quantifying the disc–fovea angle and identifying characteristic torsional patterns, fundus photography facilitates the differentiation between OTR and TNP in patients presenting with vertical diplopia. Its ease of use and compatibility with acute care settings make it especially valuable in complex or time-sensitive neurological evaluations. Although the limited number of OTR cases constrains the generalizability of our findings, the consistent torsional patterns observed suggest that fundus photography can simplify diagnostic decision-making and potentially support earlier identification of conditions such as vertebrobasilar stroke. Importantly, differentiation between conjugate vestibular-driven torsion and disconjugate muscle-specific torsion reflects distinct ocular motor control mechanisms, underscoring the relevance of torsional pattern analysis beyond simple magnitude measurements. Future prospective studies with larger cohort comparisons with established dynamic torsional measurement techniques and development of automated retinal landmark detection methods may further enhance the diagnostic precision and clinical utility of this technique.

## Figures and Tables

**Table 1:** Demographic and clinical data of patients.

Clinical Diagnosis	OTR Ocular Tilt, Skew		TNP Trochlear Nerve Palsy	
No. of patients	8		24	
Age (average)	47		48	
Gender M:F	2:6		9:15	
Vascular risk factors	HTM-2 DM-3 Chol-2 IHD-1 UK 1		HTM–5 DM–6 Chol–6 IHD–2 Healthy 13	
Smoking	3		8	
Etiology	CVA Trauma Neurosurgery SOL Cavernoma AVM Congenital Dev. delay	3 1 3 (1) (1) (1) 1 (1)	CVA Trauma Neurosurgery Demyelination Congenital Unknown	1 2 2 1 5 13

Abbreviations: HTM = hypertension; DM = diabetes mellitus; Chol = hypercholesterolemia; IHD = ischemic heart disease; UK = unknown; CVA = cerebral vascular event; SOL = space occupying lesion; AVM = arterial venous malformation; Dev. delay = development delay.

**Table 2:** Summary of statistical comparisons between OTR and TNP groups.

Variable	Group Comparison	Test Used	OTR Value	TNP Value	*p*-Value	Effect Size
Absolute total torsion	OTR vs. TNP	Mann–Whitney	25.48 ± 19.47°	23.23 ± 9.79°	Not significant	0.07
Net torsion (sum of both eyes)	OTR vs. TNP	Mann–Whitney	−11.25 ± 10.56°	23.23 ± 9.79°	<0.0001	0.68
Age	OTR vs. TNP	Mann–Whitney	47	48	Not significant	0.01
Gender (M:F)	OTR vs. TNP	Chi-squared	1:3	1:1.6	Not significant	0.09

**Table 3:** Patients with ocular tilt reaction. The table illustrates variability in interocular torsional direction within the OTR group, highlighting conjugate torsional shifts toward the same side in most cases.

	RE/LE HT	Torsion	RE Torsion	LE Torsion	Overall Torsion	Absolute Overall Torsion	Bielschowsky +/−
1	RHT	RE Intorsion	−16.94	+50.374	33.434	67.314	+ 4th step
2	RHT	RE Intorsion	−3.157	+18.214	15.057	21.371	+ Hess
3	LHT	LE Intorsion	+24.041	−8.854	15.187	32.895	+
4	LHT	LE Extorsion	−8.842	+6.194	2.648	15.036	+ Hess: BE toward left PA
5	RHT	RE Extorsion LE Intorsion	+2.920	−2.272	0.648	5.192	+
6	RHT	RE Intorsion	−5.618	+9.462	3.844	15.08	−
7	RHT no diplopia	RE Intorsion	−3.130	+10.491	7.361	13.621	N/A
8	LHT	LE Intorsion	+22.574	−10.759	11.815	33.333	N/A
Patient ages ranged from 7 to 68 years, with a mean age of 47 years. The absolute total value of the OTR group was 25.35 ± 19.55° and the sum was 11.249 ± 10.547°							

Abbreviations: RE = right eye; LE = left eye; LHT = left head tilt; RHT = right head tilt; PA = pilocytic astrocytoma.

**Table 4:** Patients with TNP.

	RE/LE	RE Torsion	LE Torsion	Overall Torsion	Absolute Overall Torsion	Bielschowsky
1	RHT	+8.38	+14.597	22.977	22.977	Positive +/−
2	RHT	+5.104	+16.168	21.272	21.272	Negative Post strabismus op (RSR recession, RIO myectomy)
3	RHT	+28.039	+24.270	52.309	52.309	Positive
4	LHT	+15.798	+7.474	23.272	23.272	Positive
5	LHT	+15.716	+10.199	25.915	25.915	Positive
6	LHT	+9.265	+11.475	20.74	20.74	Positive
7	LHT	+7.009	+9.680	16.689	16.689	Positive HESS
8	LHT	+5.563	+8.935	14.498	14.498	Positive
9	LHT	+19.265	+12.323	31.5884	31.5884	Positive
10	RHT	+4.496	+5.624	10.12	10.12	Positive
11	LHT	+22.6	+20.1	+42.70	+42.70	Positive
12	LHT	+26.767	+11.547	38.314	38.314	Positive
13	LHT	+6.281	+17.024	23.305	23.305	Positive
14	LHT	+10.24	+10.578	20.819	20.819	Positive
15	LHT	+6.772	+9.315	16.087	16.087	Positive
16	RHT	+6.198	+5.423	11.621	11.621	Positive
17	RHT	+9.554	+13.925	23.479	23.479	Positive
18	LHT	+10.414	+8.1112	18.5252	18.5252	Positive
19	LHT	+5.667	+11.862	17.529	17.529	Positive
20	LHT	+6.263	+15.50	21.763	21.763	Positive
21	LHT	+14.0	+13.1	27.10	27.10	Positive
22	RHT	+8.397	+4.467	12.864	12.864	Positive
23	RHT	+13.912	+9.567	23.479	23.479	Positive
24	LHT	+9.802	+10.78	20.582	20.582	Positive
Patient ages ranged from 5 to 79 years, with a mean age of 48 years. The mean overall torsion in the TNP group was 23.23° ± 9.8°. Torsion in all patients was noted in both eyes. In contrast to OTR, torsional values in TNP were uniformly extorsional and directionally consistent across patients, supporting a peripheral muscle-specific ocular motor mechanism. Head tilt was documented in 18 of 24 patients in the TNP group.						

Abbreviations: RE = right eye; LE = left eye; RSR = right superior rectus muscle; RIO = right inferior oblique; LHT = left head tilt; RHT = right head tilt.

## Data Availability

The data presented in this study are available on request from the corresponding author due to patient privacy.

## Abbreviations

The following abbreviations are used in this manuscript:

OCT	Optical Coherence Tomography
OTR	Ocular Tilt Reaction
TNP	Trochlear Nerve Palsy
CVA	Cerebrovascular Accident
LHT	Left Head Tilt
RHT	Right Head Tilt
